# Having seen it all: my vision of atrial fibrillation treatment in 2022

**DOI:** 10.1093/ejcts/ezx499

**Published:** 2018-03-26

**Authors:** James L Cox

**Affiliations:** 1 Center for Heart Rhythm Disorders; 2 Bluhm Cardiovascular Institute, Feinberg School of Medicine, Northwestern University, Chicago, IL, USA

**Keywords:** Atrial fibrillation, Future, Surgery

Any discussion on the interventional treatment of atrial fibrillation (AF) must begin and end with the realization that there are only 2 ways to treat cardiac arrhythmias: (i) drugs and (ii) scars. Drugs remain the predominant method of therapy for AF with more than 95% of all AF still being treated using this modality. When re-entry was first described by G.R. Mines in 1914, no electrophysiological mapping systems were available to study the details of cardiac arrhythmias, and as a result, the creation of scars in animal preparations was the primary method of studying arrhythmias. Typically, an arrhythmia would be induced in bench-top animal tissue models, and scars were created to modify the arrhythmia in hopes of yielding information regarding the nature of the induced arrhythmia. The critical component of such studies was that the investigators knew the precise location, length, width and depth of the scars. They were also certain that the scars were ‘contiguous’ with no gaps along the length of the scar and ‘uniformly transmural’ with no gaps above or below the scar.

The use of scars to ‘study’ cardiac arrhythmias in this manner was practised from 1914 until 2 May 1968 when Will C. Sealy and colleagues at Duke University first created scars to ‘treat’ a cardiac arrhythmia, namely reciprocating tachycardia resulting from the Wolff–Parkinson–White syndrome. Since that time, the evolution of electrophysiological mapping and cardiac imaging has been nothing short of revolutionary, but electrophysiological maps, no matter how sophisticated, do not come with an interpretation, and the findings may often be interpreted in more than one way. Thus, the definitive mechanism of an arrhythmia may still depend on the placement of a scar to determine the true aetiology of the arrhythmia.

One of the major problems since the advent of catheter ablation for the treatment of AF has been the validity of the conclusions drawn from the response of an arrhythmia to the creation of multiple focal scars in the atrium with the tip of a catheter in a beating, working heart. The precise location, contiguity and transmurality of such scars are unknown to the operator, but their resultant effects have been used to ‘explain’ the underlying electrophysiology of AF. Needless to say, many of those explanations have proved to be wrong, and the interventional procedures based on them have been ineffective.

Despite these problems, we find ourselves in 2017 in the enviable position of being able to cure virtually all types of cardiac arrhythmias with the minimally invasive techniques of catheter ablation. Indeed, for most arrhythmias, catheter ablation can attain essentially the same success rates that merely 20 years ago were attainable only by open-heart surgical techniques. The major exception is catheter ablation for non-paroxysmal AF, especially long-standing persistent AF. A reasonable question to ask is ‘Why have interventional cardiologists been so successful treating every clinical arrhythmia except non-paroxysmal AF?’ I believe it is because they do not have the proper tools to treat these more complex forms of AF. It has been clear for some time that while pulmonary vein isolation is adequate for the treatment of paroxysmal AF, it is manifestly inadequate for the treatment of non-paroxysmal AF. Despite this generally accepted notion, large studies such as the so-called ‘STAR-AF II Trial’ have suggested that the addition of ‘lines’ and so on adds nothing to pulmonary vein isolation in treating non-paroxysmal AF. This erroneous conclusion is a direct result of the misunderstanding of how scars modify arrhythmias. The authors of that study had no idea of the precise location, contiguity or transmurality of the ‘lines’ that were placed in their 3 study cohorts. The result is that all 3 cohorts received essentially the same LA ablation, and the wrong conclusion was reached from a poorly controlled study. This will inevitably result in numerous patients receiving an inadequate intervention for non-paroxysmal AF in the future.

So what does the future hold for the treatment of AF? Since catheter ablation is inadequate for the treatment of most non-paroxysmal AF, cardiologists and surgeons have 2 viable options in the near-term future: (i) the minimally invasive, on-pump, cryosurgical maze procedure and (ii) hybrid procedures. I view both approaches as transition procedures that will devolve into a secondary role for stand-alone AF treatment as soon as more effective tools are placed in the hands of interventional cardiologists. I believe that these tools will be catheter-based devices that will allow interventional cardiologists to create linear lesions (‘lines’) in the atria as effectively as surgeons can place them with a knife. Once such tools are available, non-paroxysmal AF will succumb to the fate of all other cardiac arrhythmias, i.e. they will become vulnerable to catheter ablation by non-surgical means.

2022? Here is what I see. More surgeons will become knowledgeable and competent at treating the vast number of patients with AF who are already entering their operating theatres for the treatment of coronary artery valve disease, mitral valve disease and aortic valve disease. Stand-alone paroxysmal AF and stand-alone non-paroxysmal AF will be treated by interventional cardiologists who will attain higher success rates using the new tools that will become available to them. The number of patients with AF who will undergo some type of intervention will increase exponentially, resulting in more arrhythmia procedures being performed by both cardiologists and surgeons. The importance of the left atrial appendage in the genesis of AF and thromboembolism, and the optimal way to manage it will be further elucidated. Most importantly, the scourge of AF and its attendant strokes will ultimately be brought under more reasonable control.

**Conflict of interest:** James L. Cox discloses a financial relationship with AtriCure.

